# Kinetic Modeling and Biological Activities of *Rosa canina* L. Pseudo-Fruit Extracts Obtained via Enzyme-Assisted Extraction

**DOI:** 10.3390/antiox14050558

**Published:** 2025-05-07

**Authors:** Zafeiria Lemoni, Styliani Kalantzi, Theopisti Lymperopoulou, Andromachi Tzani, Georgios Stavropoulos, Anastasia Detsi, Diomi Mamma

**Affiliations:** 1 Biotechnology Laboratory, School of Chemical Engineering, Zografou Campus, National Technical University of Athens, 9 Iroon Polytechniou Str, 15780 Athens, Greece; zlemoni@chemeng.ntua.gr (Z.L.); stkalantzi@hotmail.com (S.K.); 2 Products and Operations Quality Control Laboratory, School of Chemical Engineering, Zografou Campus, National Technical University of Athens, 15780 Athens, Greece; veralyb@chemeng.ntua.gr; 3 Laboratory of Organic Chemistry, School of Chemical Engineering, Zografou Campus, National Technical University of Athens, 15780 Athens, Greece; atzani@mail.ntua.gr (A.T.); adetsi@chemeng.ntua.gr (A.D.); 4 Korres S.A.—Natural Products, 57th km Athens-Lamia Road, 32011 Oinofyta Viotia, Greece; giorgos.stavropoulos@korres.com

**Keywords:** enzyme-assisted extraction (EAE), cellulolytic enzyme, pseudo-fruit of *Rosa canina* L., total phenolic content (TPC), total flavonoid content (TFC), kinetic models, biological activities

## Abstract

This study investigates the enzyme-assisted extraction (EAE) of bioactive compounds from the pseudo-fruit of the wild rose (*Rosa canina* L.), also known as rosehip, using a commercial cellulolytic enzyme preparation, Cellic^®^ CTec3 HS. The effects of extraction time, solid to liquid ratio, and enzyme loading on total phenolic content (TPC) and total flavonoid content (TFC) were evaluated. The highest yields of TPC (168.3 ± 1.1 GAE/g DM) and TFC (72.3 ± 0.8 mg CAE/g DM) were obtained at 360 min, using 1% (*v*/*v*) enzyme loading and a 6% (*w*/*v*) solid to liquid ratio. Kinetic modeling of the extraction process was performed using first-order, second-order, Peleg’s, and power law models. The power law model best described the extraction dynamics. The obtained extracts were assessed for their biological activities including antioxidant, antimicrobial, anti-aging, and anti-diabetic properties. The extract obtained under optimal extraction conditions exhibited potent tyrosinase inhibition (80%) and moderate to low inhibition of α-glucosidase (15%) and α-amylase (20%) activities. The IC_50_ for DPPH radical scavenging was 0.44 μL extract/mL while the extract exhibited significant antibacterial activity against *Escherichia coli* growth (79% inhibition). These findings suggest that the extract, obtained through EAE, has promising biological properties with potential applications in the food, pharmaceutical, and cosmetic industries.

## 1. Introduction

*Rosa canina* L. or dog rose is a wild rose species of the family Rosaceae. It is a plant that grows up to 4 m tall and is naturally distributed throughout Europe, North Africa, and Asia. The pseudo-fruit of *Rosa canina* L., commonly known as rosehip, matures from September to October and has long been known for its high content of bioactive compounds, phenolics, flavonoids, carotenoids, and mineral compounds, among others [1]. Its rich phytochemical content accounts for dog rose’s strong biological activities, such as its antioxidant, antimicrobial, anti-aging, anti-diabetic, and anti-inflammatory properties [2]. Hence, it is relevant in the prevention and treatment of many ailments along with nutraceutical, cosmetic, and pharmaceutical applications [3].

Bioactive compounds are secondary metabolites, not directly essential for basic functions, whose prolonged absence could affect growth or reproduction [4]. They are highly valuable for their potential applications in medicine, cosmetics, drugs, and other industries as key ingredients in innovative products, as they influence biological systems and lead to therapeutic effects [5]. Bioactive compounds are found in various natural sources, including plants, microorganisms, marine organisms, animal products, and even by-products or waste [6]. The bioactive compounds of plant origin are trapped within the plant cell wall and the cytosol of the plant cell. The plant cell wall is a natural nanoscale network structure composed mainly of polysaccharides such as cellulose (a linear polymer of glucose units linked by β-1,4-glycosidic linkages), hemicellulose (a non-linear heteropolymer in which main chain sugars are linked by β-1,4-glycosidic bonds) and pectin (which is, in its simplest form, a linear polymer of D-galacturonic acid linked by α-1,4-glycosidic bonds), but also present are glycoproteins and lignin (a heterogeneous polymer, phenolic in nature, derived from three lignol precursors chemically linked in various ways) [7]. Obtaining bioactive compounds is a challenging process due to the complex structure of the plant cell wall, and it is typically accomplished through extraction [8].

Extraction methods can be classified into conventional (Soxhlet, maceration, hydro-distillation, cold pressing) and non-conventional (ultrasound-assisted extraction (UAE), microwave-assisted extraction (MAE), natural deep eutectic solvent (NADES)-assisted extraction, pulsed electric field (PEF) extraction, enzyme-assisted Extraction (EAE)) [9]. Conventional methods have several drawbacks, including the use of toxic organic solvents, high energy consumption, low product quality, and safety hazards, while non-conventional ones reduce the use of toxic solvents, require milder conditions, and increase the extraction yield [9].

Extraction of the bioactive compounds from *Rosa canina* L. has been carried out by conventional methods using different solvents, such as ethanol [10], methanol [11], and water [12]. Also, non-conventional methods have been employed, such as UAE [13], microwave hydrodiffusion and gravity (MHG) [14], PEF [15], ultrasound-assisted enzymatic extraction (UAEE) [16], and supercritical carbon dioxide extraction (SFE) [17].

Among these techniques, EAE has emerged as a promising alternative, offering improved selectivity and higher yields, to overcome some of the aforementioned challenges [18]. EAE is based on the inherent ability of enzymes to catalyze reactions with high specificity and enantioselectivity in aqueous solutions at low temperatures, effectively breaking down the plant cell wall and releasing the bioactive compounds. The type of enzyme used in EAE depends largely on the composition of the plant material [19]. Compared to conventional extraction methods, EAE is a highly flexible and environmentally friendly method because it uses less energy, no organic solvents, mild conditions, and it protects compounds susceptible to heat and oxidation, all while allowing customization to the plant matrix, improving yield and selectivity [20,21]. Although EAE demonstrates promising potential, its application to *Rosa canina* L. has been underexplored.

Cellulases are widely used in industrial processes, e.g., in the food industries, wine production, and plant extraction [22]. Cellulases are a group of enzymes that catalyze the hydrolysis of cellulose. Cellulase consists of three enzymes: β-glucosidase, endo-1,4-β-D-glucanase (endoglucanase), and exo-1,4-β-D-glucanase (exoglucanase). These three enzymes are involved in the hydrolysis of cellulose by synergetic action for the complete and effective hydrolysis of cellulose [23].

The aim of this study was to present an efficient and environmentally sustainable extraction method of bioactive compounds from the pseudo-fruit of *Rosa canina* L., with emphasis on TPC, TFC, and several biological activities, including antioxidant, antibacterial, anti-aging, and anti-diabetic activities, for potential application in various industries. A major part of this study was the kinetic modeling of the extraction process, where various models were used to explore the mechanisms behind the release of bioactive compounds from the plant matrix.

## 2. Materials and Methods

### 2.1. Plant Material

The raw material used in the experiments was the dried pseudo-fruit (also referred to as rosehip) of dog rose (*Rosa canina* L.), which was kindly provided by Korres S.A.—Natural Products. It originates from plants cultivated and preserved within the Balkan Botanic Garden of Kroussia, located in northern Greece, near the mountain village of Pontokerasia in the Kilkis Regional Unit (coordinates: 41°05′ N 23°06′ E). The rosehips were collected during the first ten days of October. Dried rosehip shells (hypanthium), manually separated from the seeds, were downsized to an average particle size of <500 μm in a laboratory mill. The milled material was stored in plastic containers at room temperature until further use in the experiments.

### 2.2. Chemical and Reagents

The following reagents were used: 2,2-Diphenyl-1-picrylhydrazyl (DPPH·); absolute methanol (CH_3_OH); and Folin–Ciocalteu from Sigma-Aldrich (St. Louis, MO, USA). All other reagents were of the highest purity commercially available and were obtained from Sigma Chemical Co. (St. Louis, MO, USA).

### 2.3. Enzyme Preparations

The following commercial enzyme preparations were used: Cellic^®^ CTec3 HS which was a generous gift from Novozymes A/S (Bagsværd, Denmark), tyrosinase (EC 1.14.1.8.1) from mushroom, α-amylase (EC 3.2.1.1) from porcine pancreas, and α-glucosidase (EC 3.2.1.20) from *Saccharomyces cerevisiae*. The latter three enzymes were purchased from Sigma-Aldrich (St. Louis, MO, USA).

### 2.4. Chemical Analysis of Raw Material

Moisture, ash, crude fat, crude protein (Kjeldahl method), and total starch content were determined according to standard methods [24]. Pectic polysaccharides were determined according to Phatak et al. [25], while water-soluble materials, cellulose, hemicellulose, and acid insoluble lignin content were determined as described by Sluiter et al. [26]. Analysis was carried out in triplicate.

### 2.5. Enzyme-Assisted Extraction

The milled material was mixed with 50 mM phosphate buffer pH = 5.5 and a specific amount of enzyme was added. Extraction was performed in a thermoshaker (Thermomixer^®^, Eppendorf, Hamburg, Germany) under constant stirring conditions (1300 rpm), operating at 50 °C.

The parameters studied were the solid to liquid ratio (SLR) (2, 4, 6, and 8% *w*/*v*) and enzyme loading (0.5, 1.0 and 1.5% *v*/*v*). Samples were taken at different time intervals (30, 60, 90, 120, 240, 360, 480, 600, and 720 min); the samples were centrifuged at 10,000 rpm for 10 min at 4 °C and the supernatants were collected and stored at −18 °C until further analysis.

### 2.6. Total Phenolic Content

The total phenolic content (TPC) was determined according to the Folin–Ciocalteu method [27]. Briefly, 50 μL of the extract was mixed with 250 μL of Folin–Ciocalteu reagent and 3.95 mL distilled water. The mixture was vortexed and 750 μL of aqueous sodium carbonate solution (22% *w*/*v*) was added. The mixture was allowed to stand in the dark at room temperature for 1 h. The absorbance was measured at 755 nm and the results were expressed as mg of gallic acid equivalents (GAE) per g of dry material (DM) (mg GAE/g DM). All measurements were performed in triplicate.

### 2.7. Total Flavonoid Content

The total flavonoid content (TFC) was determined according to the aluminum trichloride method [28]. Briefly, 100 μL sample was mixed with 60 μL NaNO_2_ (5% *w*/*v* aqueous solution). After 6 min, 120 μL of AlCl_3_ (10% *w*/*v* aqueous solution) was added, the mixture was allowed to stand at room temperature for 5 min, and then 600 μL NaOH (1 M) and 1120 μL distilled water were added. The mixture was allowed to stand for 15 min at room temperature. The absorbance was measured at 510 nm. The results were expressed as mg Catechin Equivalents (CAE) per g of dry material (DM), (mg CAE/g DM).

### 2.8. Antioxidant Activity

The antioxidant activity of the extracts was determined using the colorimetric determination of DPPH radical scavenging ability [29]. In a test tube, 1950 μL DPPH solution (2.5% *w*/*v* in methanol) was added to 50 μL of extract (initial concentration Χ μL extract/mL sample) and then the same procedure was followed for samples diluted in water, with concentrations of 0.8Χ, 0.6Χ, 0.4Χ and 0.2Χ μL extract/mL sample. The samples were incubated for 30 min at room temperature in the dark and then the absorbance was measured at 515 nm. The above-mentioned procedure was repeated for a blank sample, which contained 50 μL methanol instead of the sample. All measurements were performed in triplicate. The percentage of inhibition of DPPH was calculated using the following formula:

(1) $$\mathrm{Inhibition}\;\mathrm{of}\;\mathrm{DPPH}\;\left(\%\right)=\frac{A_{\mathrm{blank}}-A_{\mathrm{sample}}}{A_{\mathrm{blank}}}\times 100$$

where A_blank_ is the absorbance of the blank samples containing methanol/DPPH and A_sample_ is the absorbance of each sample containing the extract.

The DPPH radical scavenging activity of the samples was quantified by the IC_50_ value, which represents the extract concentration that is required to reduce 50% of the initial DPPH absorbance. IC_50_ values were calculated by the linear plot of extract concentrations vs. percentage inhibition of DPPH radicals, and the results were finally expressed as μL of extract per mL of sample.

### 2.9. Antibacterial Activity

The antibacterial activity of the extracts was tested against *Escherichia coli* (*E. coli*) using the broth microdilution method [30]. The extracts were diluted in a sterile culture medium. In a 96-well plate, 50 μL of culture medium was added into every well, then 50 μL of each extract was added into the first column and mixed carefully. Then, 50 μL from the first column was transferred into the second column. The same procedure was repeated down to column number 10. Fifty μL of the bacterial suspension (10^8^ CFU/mL) was added to each well. Column 11 was used as growth control (only bacterial suspension and culture medium) and column 12 was used as sterility control (only culture medium).

The microplate was incubated at 37 °C for 24 h and then the optical density at 600 nm was read. All measurements were performed in triplicate. Antibacterial activity (inhibition of growth) was calculated as follows:

(2) $$\mathrm{Inhibition}\;\mathrm{of}\;\mathrm{growth}\;\left(\%\right)=\frac{A_{\mathrm{control}}-A_{\mathrm{sample}}}{A_{\mathrm{control}}}\times 100$$

where A_control_ is the absorbance of the growth control and A_sample_ represents the absorbance of cells containing the extracts.

### 2.10. Anti-Aging Activity (Tyrosinase Inhibition)

Inhibition of tyrosinase (EC 1.14.1.8.1), was determined using the modified dopachrome method with L-DOPA as substrate [31]. All the samples were diluted in phosphate buffer (50 mM, pH 6.8). Twenty-five μL of the sample, 40 μL of tyrosinase (200 U/mL in the same buffer), 100 μL of buffer, and 40 μL of L-DOPA (10 mM dissolved in the same buffer) were placed in a 96-well plate. Following incubation at 25 °C for 10 min, the absorbance at 492 nm was measured. All measurements were performed in triplicate. Kojic acid is a known inhibitor of tyrosinase activity and was used as the positive control (complete inhibition of tyrosinase activity).

Inhibition of tyrosinase activity was calculated as follows:

(3) $$\mathrm{Inhibition}\;\mathrm{of}\;\mathrm{tyrosinase}\;\mathrm{activity}\;\left(\%\right)=\left(1\;-\frac{A_{\mathrm{sample}}-{\;A}_{\mathrm{sample}\;\mathrm{control}}}{A_{\mathrm{negative}\;\mathrm{control}}-{\;A}_{\mathrm{blank}\;\mathrm{control}}}\right)\;\times \;100$$

where A_sample_ is the absorbance of the sample, A_sample control_ is the absorbance of the reaction mixture in the absence of substrate, A_blank control_ is the absorbance of the reaction mixture in the absence of sample and substrate and A_negative control_ is the absorbance of the reaction mixture in the absence of sample.

### 2.11. Anti-Diabetic Activity

#### 2.11.1. α-Glucosidase Inhibition

Inhibition of α-glucosidase was performed according to Tiwari et al. with some modifications [32]. The samples (100 μL) were incubated at 37 °C for 10 min with α-glucosidase (50 μL, 0.25 U/mL in 0.1 M phosphate buffer, pH 6.8). Following the addition of 50 μL of 1 mM *p*-Nitrophenyl-α-D-glucopyranoside solution in 0.1 M phosphate buffer, pH 6.8, the mixture was incubated for an additional 5 min. Absorbance at 405 nm was measured against a blank solution (the sample was replaced with buffer). All measurements were performed in triplicate. Inhibition of α-glucosidase activity was calculated as follows:

(4) $$\mathrm{Inhibition}\;\mathrm{of}\;\alpha -\mathrm{glucosidase}\;\mathrm{activity}\;\left(\%\right)=\frac{A_{\mathrm{blank}}-A_{\mathrm{sample}}}{A_{\mathrm{blank}}}\times 100$$

where A_blank_ is the absorbance of the blank and A_sample_ is the absorbance of the sample

#### 2.11.2. α-Amylase Inhibition

Inhibition of α-amylase activity was performed using Caraway–Somogyi iodine/potassium iodide (IKI) method [33] with some modifications. The sample (25 mL) was mixed with α-amylase solution (50 μL, 0.5 U/mL dissolved in phosphate buffer pH 6.9 with 6 mM sodium chloride) in a 96-well plate and incubated for 10 min at 37 °C. After pre-incubation, the reaction was initiated with the addition of starch solution (50 μL, 0.025%, dissolved in the same buffer), and the mixture was incubated at 37 °C for an additional 10 min. The reaction was terminated by adding 1 M HCl solution (25 μL). This was followed by the addition of the iodine–potassium iodide solution (100 μL). The absorbance was read at 630 nm. All measurements were performed in triplicate. The inhibition of α-amylase activity was calculated as follows:

(5) $$\mathrm{Inhibition}\;\mathrm{of}\;\alpha -\mathrm{amylase}\;\mathrm{activity}\;\left(\%\right)=\left(1-\frac{A_{s_{s}}-A_{s_{b}}}{A_{s_{c+}}-{\;A}_{s_{c-}}}\right)\times 100$$

where $A_{s_{s}}$ is the absorbance of the sample, $A_{s_{b}}$ is the absorbance of the reaction mixture in the absence of enzyme, $A_{s_{c+}}$ is the absorbance of the reaction mixture in the absence of the sample (100% enzyme activity), $A_{s_{c-}}$ is the absorbance of the mixture without sample and enzyme (0% enzyme activity).

### 2.12. Kinetic Modeling

Mathematical modeling offers valuable insights for scaling up extraction processes by identifying the optimal conditions. Several mathematical models have been employed to describe the extraction of bioactive compounds from plant materials, including the first-order, second-order, Peleg’s, and power laws [34].

#### 2.12.1. First-Order Model

According to Harouna-Oumarou et al. [35] solid to liquid extraction can be considered as the opposite operation of adsorption (mass transfer of solute between a solid phase and a solvent). The equation commonly applied to adsorption can also be adapted for the extraction process, where the kinetics is frequently described by a first-order reaction. Recently, a first-order kinetic model was evaluated for the extraction of *Vernonia cinerea* leaf [36]. The first-order kinetic model can be written as per Equation (6). It is assumed that the first-order rate equation is correlated with the idea of a linear driving force [34].

(6) $$\frac{{\mathrm{dC}}_{t}}{\mathrm{dt}}=k_{1}\;\left(C_{s}-C_{t}\right)$$

where $C_{t}$ is the extraction capacity at different extraction times t, (mg/g), k_1_ is the first-order extraction rate coefficient, (min^−1^), and $C_{s}$ is the concentration of bioactive compounds at equilibrium (mg/g).

By integrating Equation (6) with the boundary conditions $C_{t}$ = 0 to $C_{t}\;$ and t = 0 to t, the following exponential equation can be obtained for the description of the extraction process:

(7) $$C_{t}=C_{s}\left(1-e^{-k_{1}t}\right)$$

#### 2.12.2. Second-Order Model

The mechanism of a second-order kinetic model indicates that the extraction occurs in two simultaneous processes. The quantity of extracted bioactive compounds improves with time in the beginning and declines gradually with time until the extraction process ends [34]. The second-order kinetic model can be written in the differential form as follows:

(8) $$\frac{{\mathrm{dC}}_{t}}{\mathrm{dt}}=k{\left(C_{s}-C_{t}\right)}^{2}$$

where k is the second-order extraction rate constant (g/mg·min), $C_{s}$ is the extraction capacity, representing the concentration of bioactive compounds at equilibrium (mg/g), $C_{t}$ is the concentration of bioactive compounds at a given extraction time (mg/g), and t is the extraction time (min).

Equation (8) was integrated, applying the boundary conditions $C_{t}$ = 0 to $C_{t}$, and t = 0 to t (Equation (9)):

(9) $$C_{t}=\frac{{C_{s}}^{2}\mathrm{kt}}{1+C_{s}\mathrm{kt}}$$

#### 2.12.3. Peleg’s Model

Peleg’s model is a semi-empirical kinetic model introduced by Peleg, widely used to explain the extraction curves of biological materials from plant sources because of their similarity to the shape of sorption curves. According to Peleg’s model, the extraction takes place in two stages: at the start, it is first order, and it then moves down to zero order. The model describing the sorption isotherms is as follows [34]:

(10) $$C_{t}=C_{0}+\frac{t}{K_{1}+K_{2}t}$$

where $K_{1}$ is the Peleg’s rate constant (min·g/mg), $K_{2}$ is the Peleg’s capacity constant (g/mg), $C_{t}$ is the concentration of bioactive compounds at any time (mg/g), and $C_{0}$ is the initial concentration of bioactive compounds (mg/g).

As the initial concentration of the target bioactive compounds is zero, the term $C_{0}$ can be omitted from Peleg’s equation (Equation (11)):

(11) $$C_{t}=\frac{t}{K_{1}+K_{2}t}$$

At the very beginning of the extraction, the term, $K_{2}t$ in Equation (11) is small enough and can be considered zero, so the solute concentration is as follows:

(12) $$C_{t}=\left(\frac{1}{K_{1}}\right)t$$

and when t tends to infinity the system reaches the equilibrium

(13) $$C_{\mathrm{eq}}=\frac{1}{K_{2}}$$

where $C_{\mathrm{eq}}$ is the equilibrium concentration (mg/g) as t tends to infinity.

#### 2.12.4. Power Law Model

The power law kinetic model is a mathematical model used to describe the extraction mechanism by the diffusion of solute through a non-swelling material [34]. The power law model is described by Equation (14):

(14) $$C_{t}={\mathrm{Bt}}^{n}$$

where $C_{t}$ is the concentration of bioactive compounds in the solution at any time (mg/g), B is a constant related to the extraction rate (mg/g/min), n is the diffusional exponent, indicative of transport mechanisms, and t is the extraction time (min). The value of n is lower than 1 for the extraction of plant materials [37].

### 2.13. Statistical Analysis

The data were confirmed to be normally distributed and to exhibit homogeneity of variances according to Shapiro–Wilk Test and Levene’s Test, respectively. Analysis of variance (ANOVA) and Tukey’s HSD test were used to assess the possible differences among the means. The results were expressed as mean values with the standard deviation (SD) of three independent measurements (n = 3) and significance was assumed at *p* < 0.05. Pearson’s correlation analysis was performed using Microsoft Excel, setting the level of statistical significance at *p* < 0.05. Non-linear regression was used to determine the parameters of the kinetic models from the experimental data, using SigmaPlot software (Version 12.5). The consistency between experimental and predicted values was assessed using the correlation coefficient (R^2^ and adjusted R^2^) and the normalized root mean square deviation (NRMSD) as evaluation metrics.

## 3. Results

### 3.1. Composition of the Hypanthium of Pseudo-Fruit of Rosa canina L.

In order to develop an efficient enzyme-assisted extraction (EAE) method, it is necessary to know the composition of the plant material. The pseudo-fruits, which are called rosehips, are aggregate fruits consisting of several achenes (the actual seed-containing fruits of rosehips) enclosed by an enlarged, red-to-orange-colored, fleshy floral cup (hypanthium). The pseudo-fruits are comprised of 30–35% seeds and 65–70% hypanthium [1]. In the present study, bioactive compounds were extracted from the hypanthium of rosehips. Table 1 illustrates the composition of the hypanthium. The moisture content of the raw material was found to be 6.7 ± 0.3% (*w*/*w*), while no starch was detected. Glucose and fructose were found among the water-soluble compounds. Ognyanov et al. (2022) reported that the main sugars detected in the rosehips of *R. canina* L. (cultivated in Smolyan, Rhodope Mountains, Bulgaria) were glucose and fructose, accounting for 80% of total free sugar, while galactose and xylose were detected in smaller quantities [38].

The polysaccharide content (pectin, cellulose, and hemicelluloses) of the hypanthium makes up approximately 32% (*w*/*w*, dry basis) of the material, while the crude fat content is about 5.4% (*w*/*w*, dry basis). It should be noted that the oil content of the hypanthium is four times lower than that found in the seeds [1].

The polysaccharide, protein, and crude fat content of the hypanthium (Table 1) were found in the range reported in the literature. More specifically, the cellulose content of the pseudo-fruits of different wild rose varieties cultivated in different geographical areas ranged from 2.1 to 9.7%, while the fat content ranged from 0.7 to 5.4% [39,40,41,42]. The protein content depends on the stage of fruit ripening, climatic conditions, and is even correlated with the altitude at which the plants grow, and it ranged from 2.3 to 8.5% [39]. Finally, pectin content reported in literature, expressed as uronic acids, ranged from 9.6 to 10.5% [39]. In general, the differences observed in the composition of the fruits of *R. canina* L. could be attributed to environmental conditions (the region where the plant grows), as well as to the harvest season and storage conditions. Since cellulose and hemicellulose are among the key structural components of the rosehip’s cell wall, EAE using the appropriate hydrolytic enzymes can be expected to have a beneficial effect in the recovery of bioactive compounds. Cellic^®^ CTec3 HS contains proficient cellulase components boosted by proprietary enzyme activities, including advanced AA9 molecules, improved β-glucosidases, as well as a new array of hemicellulase activities [43].

### 3.2. Total Phenolic Content (TPC)

**Figure 1:**
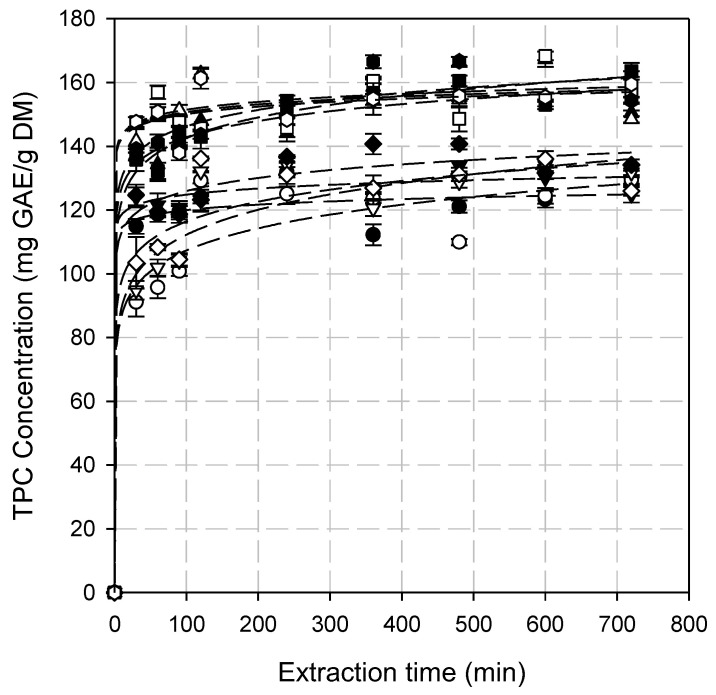
The extraction of total phenolic content (TPC) from the hypanthium of the pseudo-fruit of *Rosa canina* L. at different combinations of SLR and enzyme loading. The dashed lines represent the data fitting to the power law model. Symbols include the following: SLR (%, *w*/*v*)/enzyme loading (%, *v*/*v*): (●) 2/0.5, (○) 4/0.5, (▲) 6/0.5, (△) 8/0.5, (▼) 2/1.0, (▽) 4/1.0, (■) 6/1.0, (☐) 8.0/1.0, (♦) 2/1.5, (◇) 4/1.5, (⬢) 6/1.5 and (◈) 8/1.5.

Analysis of the parameters that affect the extraction process is crucial, as it helps reduce resource consumption (such as time and energy) while ensuring optimal extraction efficiency. The experimental data indicated that enzyme loading and SLR affected TPC extraction. Analysis of variance (ANOVA) verified that all the parameters examined are statistically significant (*p* < 0.05). The results suggest, regardless of the parameter combination, two extraction stages, including a rapid release of phenolic compounds during the first 30 min of the process (washing phase), followed by a decrease in the extraction rate after 60 min, where the diffusion phenomena become dominant. During the second stage, the extraction curves exhibit an asymptotic trend, suggesting minimal variation in extraction yield over time (Figure 1). Additionally, the kinetic behavior differs among various combinations of SLR and enzyme loading, particularly during the initial washing phase, when the TPC concentration in the solvent phase increases rapidly (Figure 1). The higher the SLR and enzyme loading, the higher the initial extraction rate. This could be attributed to faster disruption of the cell wall structure at higher enzyme loadings resulting in the enhanced release of bioactive compounds [44].

**Figure 2:**
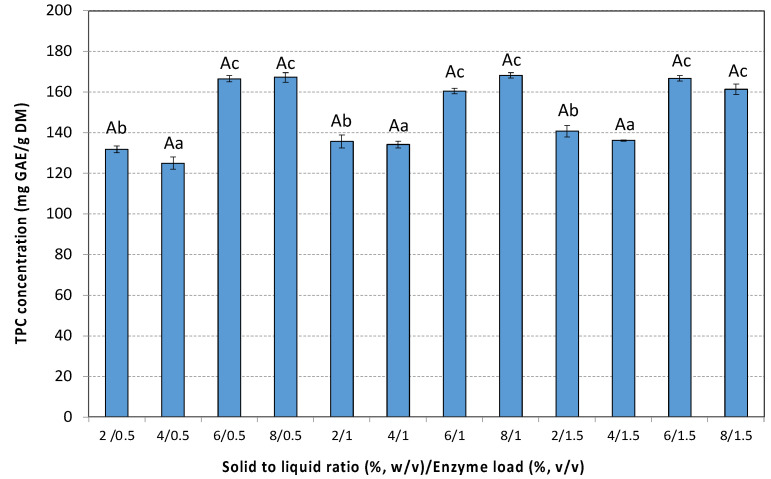
TPC extracted with the aid of cellulolytic enzyme from the hypanthium of the pseudo-fruit of *Rosa canina* L. at different combinations of SLR and enzyme loadings. The data are expressed as mean values ± SD and were analyzed using ANOVA to assess the effect of each factor, followed by Tukey’s HSD post-hoc analysis test. The superscript letters indicate significant differences (*p* < 0.05) within the levels of each specific factor. Lowercase letters are used to denote differences within the solid to liquid ratio, and uppercase letters are used to denote differences within the enzyme load. Values with different superscript letters are significantly different from each other (*p* < 0.05).

The optimal extraction time ranges from 120 to 480 min depending on the SLR as well as on the enzyme loading. Tuckey’s test, however, showed that the differences between TPC concentrations for extraction times higher than 360 min were not statistically significant (*p* < 0.01). Increasing SLR, for all enzyme loadings tested, resulted in higher TPC yields. However, statistically significant differences in TPC yields were observed up to a 6% (*w*/*v*) rosehip concentration (*p* < 0.05). A further increase in SLR does not provide additional benefits. Enzyme loading influenced the extraction process; however, no significant differences were observed between 1% and 1.5% *v*/*v* for all SLRs tested (Figure 2).

Maximum TPC yield (168.3 ± 1.1 mg GAE/g DM) was observed in 360 min at a 6% (*w*/*v*) rosehip concentration, supplemented with 1.0% (*v*/*v*), Cellic^®^ CTec3 HS. A control experiment was carried out under identical conditions without enzyme addition, yielding a TPC of 91.6 ± 1.8 mg GAE/g DM. It is evident that EAE resulted in a TPC yield approximately 45% higher, highlighting the substantial improvement in extraction efficiency driven by enzymatic hydrolysis.

According to the literature data on the EAE of different plants, enzyme loading ranged from 0.01 to 10% (*w*/*w*). In general, increasing enzyme loading leads to higher extraction yields. However, beyond a certain threshold—determined by the enzyme type and biomass characteristics—there may be no noticeable improvement or even a decline in extraction efficiency [45]. Moreover, applying low enzyme loadings can reduce the overall extraction cost, given the high cost of enzymes.

Our results compare favorably with TPC yields reported in the literature that applied conventional or non-conventional (green) extraction methods (Table 2). The majority of the studies applied maceration, a conventional solvent extraction method, using water, ethanol, methanol, or different aqueous ethanol or methanol solutions. TPC yields ranged from 2.6 to 290 mg GAE/g DM using different rosehip concentrations, in the range of 2 to 50% (*w*/*v*), depending on extraction times (2 min to 72 h). For instance, extraction of rosehips for 1 h with a 50% (*v*/*v*) hydroethanolic solution yielded 69.4 mg GAE/100 g DM [46], while using a 40% (*v*/*v*) hydroethanolic solution under the same conditions (rosehip concentration and extraction time) resulted in 21.6 mg GAE/100 g DM [47]. One of the highest TPC yields, 290 mg GAE/g DM, was reported by Kerasioti et al., who used methanol as an extraction solvent, a high substrate concentration (50%, *w*/*v*), as well as an extraction time of 48 h [48]. A high extraction time (72 h) was also applied by Nadpal et al., who used a moderate substrate concentration and 80% (*v*/*v*) aqueous methanol solution, resulting in 50.3 mg GAE/g DM [12]. By changing the extraction solvent to water, keeping the substrate concentration constant, and lowering the extraction time, 61 mg GAE/g DM were obtained by the same authors [12]. The maceration process as mentioned by Jha and Sit might take hours to weeks for some plant materials, making it a very slow process, suitable for extracting heat labile compounds [49]. Time of extraction, however, is among the important parameters of any extraction process. In general, prolonged extraction times increase both energy and operational costs and may lead to the degradation of bioactive compounds.

Green extraction techniques such as ultrasound-assisted extraction (UAE) exhibited reduced extraction times, yielding around 47.23 mg GAE/g DM obtained in approximately 1.35 h under controlled conditions [13], though its performance can be sensitive to factors such as the solvent composition, temperature, and extraction duration, as demonstrated Moldovan et al. [50] (Table 2). Microwave hydrodiffusion and gravity (MHG), has also been successfully applied, achieving relatively high TPC yields (200 mg GAE/g DM), with markedly shorter processing times; however, the rapid and localized heating associated with this technique may compromise the integrity of sensitive compounds [14]. Supercritical fluid extraction (SFE), which employs supercritical CO_2_, is considered a promising green alternative; however, it showed relatively low total phenolic content (TPC) yields, approximately 5.12 mg GAE/g dry matter (DM) [17].

In this context, the EAE technique applied herein exhibits several advantages over conventional and alternative green methods. Operating under mild conditions, EAE minimizes the thermal degradation of heat-sensitive compounds while enzymatic hydrolysis effectively disrupts cell wall matrices to enhance phenolic release. This approach combines high extraction efficiency with lower energy consumption, underscoring its novelty and industrial scalability for the sustainable recovery of bioactive compounds [13,14,16,50].

**Table 2:** A summary of recent studies on the extraction of TPC and TFC from *R. canina* L. using various extraction methods.

Extraction Method	Solvent System	Extraction Time (h)	SLR (%, *w*/*v*)	Temperature (°C)	TPC (mg GAE/g DM)	TFC (mg/g DM)	References
Maceration	EtOH:H_2_O (1:1, *v*/*v*)	1	5	20	69.40	-	[46]
	EtOH:H_2_O (1:1, *v*/*v*)	20	10	25	0.22	-	[10]
	Boiling water	1	10	25	61.00	1.14 **	[12]
	EtOH:H_2_O (80:20, *v*/*v*)	72	10	25	50.30	0.63 **	[12]
	Methanol	0.03	12	4	16.61	-	[11]
	Methanol	48	50	-	290	118 *	[48]
	MeOH:H_2_O (60:40, *v*/*v*)	-	-	5	2.15	0.02 *	[51]
	MeOH:H_2_O (80:20, *v*/*v*)	0.33	25	25	177	4.85 **	[52]
	MeOH:H_2_O (1:1, *v*/*v*)	2	10	25	48.7	6.7	[53]
	MeOH:H_2_O:HCOOH (50:48.5:1.5, *v*/*v*/*v*)	-	2	4	31.08	-	[54]
	n-hexane:acetone:EtOH (2:1:1, *v*/*v*/*v*) plus 50 mg/L butylated hydroxytoluene	0.25	-	5	2.98	1.45 *	[55]
	EtOH:H_2_O (40:60, *v*/*v*)	0.83	50	25	21.61	1.8 ***	[47]
UAE	EtOH:H_2_O (40:60, *v*/*v*)	1.35	-	25	47.23	-	[13]
	EtOH:H_2_O (70:30, *v*/*v*)	0.50	-	50	24.00	1.4 **	[50]
UAEE	0.6 U/mL pectinase, 0.3 U/mL cellulase, 0.3 U/mL hemicellulase (2:1:1, *v*/*v*/*v*) in phosphate buffer pH 5.6	0.83	5	50	32.64	-	[16]
PEF	Distilled water	0.33	5	24	70	-	[15]
MHG	Distilled water	15 min pretreatment before MHG	-	8	200	190 *	[14]
SFE	Supercritical CO_2_	0.5	-	25	5.12	1.95 *	[17]
EAE	1.0% *v*/*v* Cellic Ctec3	6	6	50	168.3	72.3 *	Present study

* CAE: Catechin equivalents, ** QE: Quercetin equivalents, *** RE: Rutin equivalents.

Differences in TPC contents reported in the literature apart from the extraction method could also be attributed to different *R. canina* ecotypes. For instance, Bozhuyuk et al. reported that the TPC of 20 ecotypes naturally grown in Kars province, located in Eastern Anatolia, Turkey, ranged from 398 to 511 mg GAE/100 g of fresh sample [56].

### 3.3. Total Flavonoid Content (TFC)

**Figure 3:**
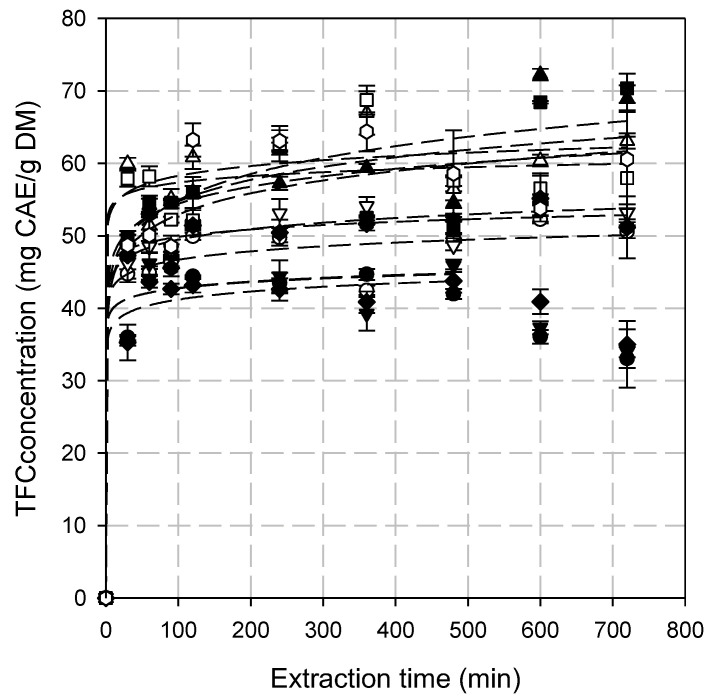
The extraction of total flavonoid content (TFC) from the hypanthium of the pseudo-fruit of *Rosa canina* L. at different combinations of SLR and enzyme loadings. The dashed lines represent the data fitting to the power law model. Symbols include the following: SLR (%, *w*/*v*)/enzyme loading (%, *v*/*v*): (●) 2/0.5, (○) 4/0.5, (▲) 6/0.5, (△) 8/0.5, (▼) 2/1.0, (▽) 4/1.0, (■) 6/1.0, (☐) 8.0/1.0, (♦) 2/1.5, (◇) 4/1.5, (⬢) 6/1.5 and (◈) 8/1.5. For the combinations 2/0.5, 2/1.0, and 2/1.5, the model fitting was conducted for up to 480 min.

Across all combinations, TFC yields rapidly increased within the first 30 min of extraction (*p* < 0.001). After the initial burst, the TFC yield continued to rise, but at a slower rate, and eventually stabilized, as in the case of TPC. This suggests that most flavonoids had already been extracted, and equilibrium is reached between the solid and liquid phases. At a prolonged extraction time (>360 min) a slight decline or fluctuation in TFC yield was observed in some conditions (Figure 3). The ANOVA of the TFC yields indicated that all parameters are statistically significant (*p* < 0.05).

Extraction times higher than 360 min did not increase significantly the TFC yield, as confirmed by the statistical analysis (Figure 3). It seems that the optimal extraction time is found between 240 and 360 min, depending on SLR and enzyme loading. At the lowest SLR and all enzyme loadings, TFC yield decreased significantly over time, probably due to gradual flavonoid degradation, which has also been reported in the literature. The extent of degradation depends on the extraction method and the structural characteristics of the compound [57,58]. The optimal extraction time is the result of two conflicting factors: (a) the higher release of TPC and TFC due to increased cell wall degradation, and (b) the higher susceptibility of released TPC and TFC to degradation. The ideal duration is determined by the balance between these factors, which is influenced by the treatment temperature, as it affects both the release and degradation of TPC and TFC [45].

**Figure 4:**
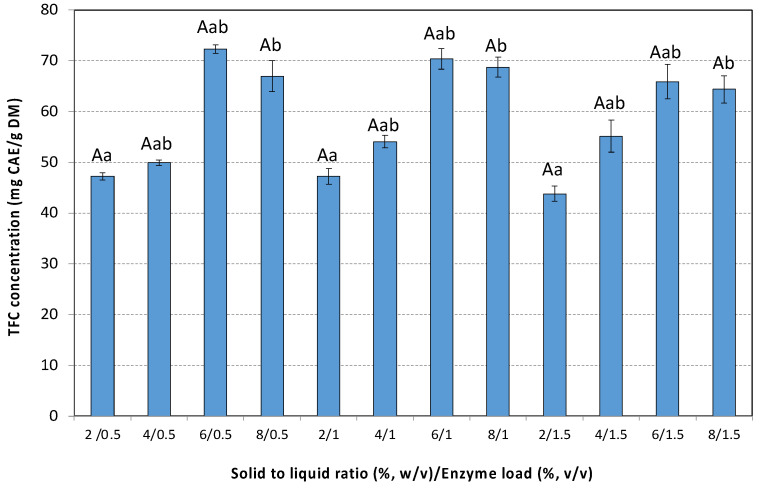
TFC extracted with the aid of cellulolytic enzyme from the hypanthium of the pseudo-fruit of *Rosa canina* L. at different combinations of SLR and enzyme loadings. The data are expressed as mean values ± SD and were analyzed using ANOVA to assess the effect of each factor, followed by Tukey’s HSD post-hoc analysis test. The superscript letters indicate significant differences (*p* < 0.05) within the levels of each specific factor. Lowercase letters are used to denote differences within the solid to liquid ratio, and uppercase letters are used to denote differences within the enzyme load. Values with different superscript letters are significantly different from each other (*p* < 0.05).

The SLR had a notable influence on TFC yields. Increasing the rosehip concentration up to 6% (*w*/*v*) led to higher TFC yields across all enzyme loadings tested. A further increase in SLR at 8% (*w*/*v*) does not improve the TFC yields (Figure 4).

Increasing enzyme loading above 0.5% (*v*/*v*) does not improve the extraction process. A decrease in TFC yield was observed at the highest enzyme loading (1.5% *v*/*v*) (Figure 4). Liu et al. investigated the EAE of flavonoids from *Acanthopanax senticosus* and reported that an increase in enzyme loading led to a decrease in TFC [59]. This reduction was attributed to the enhanced dissolution of impurities, such as polysaccharides and proteins, which interfered with flavonoid dissolution and lowered their concentration in the solution. The maximum TFC yield (72.3 ± 0.8 mg CAE/g DM) was achieved using 6% *w*/*v* rosehip treated with 1.0% *v*/*v* Cellic^®^ CTec3 HS (Figure 4). The control experiment conducted under identical conditions without enzyme addition yielded a TFC of 40.9 ± 4.2 mg CAE/g DM. The TFC yield achieved by applying EAE was 43% higher compared to the control experiment, indicating the marked improvement in extraction efficiency due to enzymatic hydrolysis. A review of the literature revealed that there are considerably fewer studies reporting TFC in *Rosa canina* L. extracts compared to TPC. Many of the available studies express TFC as equivalents of quercetin or rutin, while those of the present study are expressed as catechin equivalents, making direct comparisons challenging (Table 2). Our results compare favorably with those reported in the literature. Applying maceration with a 60:40 (*v*/*v*) methanol/water mixture, 2.45–10.2 mg CAE/g DM were obtained [51], while changing the extraction solvent to a mixture of n-hexane, acetone, ethanol, (2:1:1, *v*/*v*/*v*), supplemented with 50 mg/L butylated hydroxytoluene, resulted in a significantly higher TFC yield (1.5 mg CAE/g DM) [55]. A similar yield was reported during rosehip extraction with supercritical CO_2_ [17]. The highest yield reported in the literature is 190 mg CAE/g DM obtained by applying MHG [14].

### 3.4. Kinetic Modeling of the Extraction Process

Various mathematical models have been proposed to describe the solid to liquid extraction of bioactive compounds from plants [34]. Selecting the optimal equation to model potential industrial applications is critical from an engineering standpoint, as it minimizes processing errors, enhances the accuracy of the process, and improves the final product’s quality.

To determine the most suitable model for describing EAE, experimental data of TPC and TFC extraction under different SLRs and enzyme loadings were fitted to the first-order, second-order, Peleg’s, and power law models and statistically analyzed.

The kinetic models were assessed based on their correlation coefficients (R^2^ and adjusted R^2^) and normalized root means squared deviation (NRMSD). The constants of each model as well as the regressed statistical parameters are summarized in Table 3 and Table 4. Higher R^2^ and adjusted R^2^ values and low NRMSD values indicate a better fit of the model, demonstrating its strong alignment with the experimental data [44].

As shown in Table 3, all models have high R^2^ values (>0.940), with the second-order and power models having the highest R^2^ (0.954 to 0.986 for second-order and 0.942 to 0.994 for power model) and adjusted R^2^ values (0.948 to 0.984 for second order and 0.935 to 0.993 for power). NRMSD (%) values vary (2.55 to 17.45%) between models, indicating differences in model accuracy. Typically, a model’s fit is characterized as very good if the NRMSE is ≤10%, as better within the range of 10 to 20%, as acceptable between 20% and 30%, and as poor if it exceeds 30%. Based on the NRMSD values, all models except one could be considered very good (Table 3). However, the second-order as well as the power law model seemed to be the most accurate ones compared to Peleg’s and the first-order model, as they have the lowest NRMSD in most cases (2.5 to 8.2% for the power model and 3.4 to 7.2% for second-order model). On the basis of the R^2^, adjusted R^2^, and the NRMSD values of the studied models for TPC extraction, their goodness of fit to the experimental data in descending order is as follows: power law ≈ second order (lowest NRMSD, highest R^2^, and highest adjusted R^2^), followed by first order and Peleg’s.

The power law model expresses the relationship between the amount of extracted compound and the extraction time through a power function. The B value reflects the overall speed of the extraction process while the diffusional exponent n indicates the transport mechanism. The calculated value of n in the power law model was found in the range of 0 < n < 1 (Table 3), suggesting that even with the enzymatic breakdown of cell walls, the overall extraction rate is decreasing over time, indicating that diffusional limitations are becoming increasingly significant [37]. Based on the data presented in Table 3, it can be concluded that higher SLR increases extraction yield (B) but slows down the process (low n), while higher enzyme loading at moderate SLR enhances the initial extraction rate (higher n). High B values combined with low n values (e.g., 8% *w*/*v* combined with 1% *v*/*v* enzyme loading) suggest that while the extraction capacity is high, the process is highly diffusion-limited, while lower B values with higher n values (e.g., 4% *w*/*v* combined with 1% *v*/*v* enzyme loading) suggest that extraction is more influenced by washing effects and less by diffusion resistance. A 6% SLR with moderate enzyme loading (~1%) appears to be a balanced condition, where both washing and diffusion effects contribute efficiently to the extraction process.

Table 4 depicts the constants and statistical parameters for the models describing TFC extraction. Generally, as in the case of modeling TPC extraction, all models have high R^2^ values. More specifically, the power model exhibited the highest R^2^ values, as well as adjusted R^2^ ones, followed by Peleg’s, the first-order, and the second-order model, with small differences. The NRMSD (%) values varied between a minimum of 2.73% (first-order model) and a maximum of 17.57% (second-order model). Overall, the power and Peleg’s models exhibited the lowest average NRMSD values. The ranking of the models for TFC extraction in descending order could be as follows: the power law ≈ Peleg’s (lowest NRMSD, highest R^2^, and highest adjusted R^2^), the first-order, and the second-order model. The data in Table 4 show that the higher SLRs (8%, *w*/*v*) generally result in increased B values, indicating greater extraction efficiency, but often correspond to lower n values, suggesting a diffusion-controlled process. Conversely, moderate SLR (6%, *w*/*v*) with higher enzyme loadings results in relatively higher n values, indicating faster initial extraction kinetics.

Various mathematical models have been widely applied to study the extraction of bioactive compounds from different matrices. The second-order kinetic model was used to evaluate the EAE of rosemary leaves, demonstrating its effectiveness in describing the process [60]. Experimental data on the TPC extracted from wheat and oat bran by UAE showed a good fit with Peleg’s model [61]. The UAE of bioactive compounds from Camu-camu (*Myrciaria dubia*) fruits was assessed using four mathematical models—parabolic diffusion, power law, hyperbolic, and pseudo-second-order—where the hyperbolic and pseudo-second-order models provided the best fit [37].

The efficiency of different extraction processes, including conventional solid to liquid extraction and UAE, has also been evaluated for TPC extraction from lentil seed coats. Among the four experimental kinetic models tested—parabolic diffusion, power law, hyperbolic, and Elovich—the power law model demonstrated the best fit [62]. Deep eutectic solvents (DES) have been applied in UAE for extracting TPC from coffee silver skin, where the power law model showed the highest accuracy among the three kinetic models examined, including Elovich and two-site models [63].

A broader analysis of kinetic modeling in TPC extraction from fresh and distilled grape marc incorporated six different models: parabolic diffusion, power law, Weibull’s equation, Elovich’s equation, second-order rate, and two-site kinetic models. The results indicated that the two-site model provided the best fit [44]. Further studies on the kinetics of flavonoid extraction from peanut shells compared the phenomenological model with Peleg’s model, revealing that the phenomenological model exhibited the highest consistency with the experimental data [64]. Among the models used in the present study, the power law and Peleg’s models are predominantly empirical models. In contrast, first-order and second-order models are derived from chemical kinetics principles and possess a theoretical basis regarding reaction rates and mass transfer. Kinetic models can serve as a valuable tool for the description of EAE. Nevertheless, they are subject to several limitations when applied to complex, heterogeneous plant matrices. They assume homogeneous systems and neglect the structural heterogeneity of plant matrices, which can significantly influence enzyme–substrate interactions and mass transfer dynamics. Additionally, enzyme deactivation over time, or inhibitory compounds released during extraction, are not taken into consideration.

### 3.5. Biological Activities

*Rosa canina* L. is widely recognized for its rich and complex phytochemical profile, which contributes significantly to its therapeutic potential, making it an important natural ingredient in the cosmetic, pharmaceutical, and nutraceutical industries.

Extracts that exhibited the maximum TPC concentration were assessed for their antibacterial and antioxidant activity as well as their potential to inhibit the activity of tyrosinase (anti-aging activity), α-amylase, and α-glucosidase (anti-diabetic activity).

#### 3.5.1. Antibacterial Activity

The bioactive compounds in plant extracts exhibit diverse mechanisms of action on bacterial cells, such as the degradation of the cell wall, destabilization of the cytoplasmic membrane, inhibition of intracellular enzymes involved in metabolic processes, and interference with nucleic acids, ultimately inhibiting replication and transcription. Polyphenols show antibacterial activity against a large number of bacteria (including Gram-positive and Gram-negative bacteria) and fungi. The structure of polyphenols, mainly the presence and position of hydroxyl functional groups, are relevant to their antibacterial activity. Gram-negative bacteria are generally more resistant to antimicrobial agents than gram-positive ones [65].

**Figure 5:**
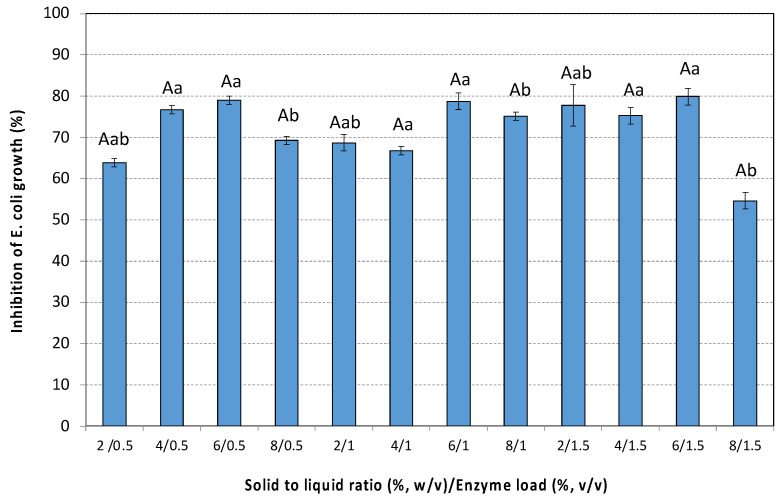
The antibacterial activity (inhibition of *E. coli* growth) by the extracts of the pseudo-fruit of *Rosa canina* L. The data are expressed as mean values ± SD and were analyzed using ANOVA to assess the effect of each factor, followed by Tukey’s HSD post-hoc analysis test. The superscript letters indicate significant differences (*p* < 0.05) within the levels of each specific factor. Lowercase letters are used to denote differences within the solid to liquid ratio, and uppercase letters are used to denote differences within the enzyme load. Values with different superscript letters are significantly different from each other (*p* < 0.05).

The activity of the extracts against the growth of the gram-negative bacterium *Escherichia coli* is shown in Figure 5. The antibacterial activity of the extracts ranged from 55 ± 2% to 80 ± 2% depending on the extraction conditions. Generally, an increase in SLRs up to 6% (*w*/*v*), as well as in enzyme loading up to 1% (*v*/*v*) resulted in extracts that exhibited increased inhibition of *E. coli* growth. The highest SLR (8%, *w*/*v*) combined with the highest enzyme loading (1.5%, *v*/*v*) does not improve the antibacterial activity of the extract. The extract that showed the highest antibacterial activity (80 ± 2%) was obtained from the treatment of 6% (*w*/*v*) rosehips with 1.5% (*v*/*v*) Cellic^®^ CTec3 HS.

The relationship between antibacterial activity and TPC/TFC was evaluated with Pearson’s correlation analysis, a statistical method used to measure the strength and direction of a linear relationship between two continuous variables. It is represented by the Pearson correlation coefficient (r), which ranges from −1 to +1. A correlation between the variables can be (a) positive (r > 0): as one variable increases, the other also increases, (b) negative (r < 0): there is an inverse relationship between the variables, or (c) there is no correlation between the variables (r ≈ 0). The strength of correlation could be (a) strong (0.70 < r < 1.00 or −0.70 < r < −1.00) when the two variables have a clear relationship, either positive or negative, (b) moderate (0.30 < r < 0.70 or −0.30 < r < −0.70) when there is a noticeable positive or negative relationship, but other factors may also influence it, or (c) weak (0.00 < r < 0.30 or −0.30 < r < 0.00) when the relationship between the variables is weak or negligible [66].

Both TPC (r = 0.127, *p* > 0.05) and TFC (r = 0.102, *p* > 0.05) show weak positive correlations with antibacterial activity, meaning that as TPC or TFC increases, antimicrobial activity tends to increase slightly, but the relationship is not strong. Additionally, the high *p*-values (>0.05) indicate that these correlations are not statistically significant. Similarly to the present study, Ispiryan et al. observed moderate, weak, or very weak correlations between the antibacterial activity and TPC of *Rubus idaeus* L. (raspberry) extracts, suggesting that although polyphenols contribute to antimicrobial effects, they are not the only influencing factors [67]. The antibacterial activity of plant extracts is affected by various factors including the structure of the polyphenols, the presence of other bioactive compounds such as alkaloids, tannins, or terpenes which may have stronger effects, the synergistic or antagonistic effect between polyphenols, and the other plant compounds.

Numerous studies have demonstrated the antibacterial properties of *Rosa canina* pseudo-fruit extracts against *E. coli*. Montazeri et al. found that the aqueous extract inhibited *E. coli* growth, with a Minimum Inhibitory Concentration (MIC) of 25 mg/mL [68]. In contrast, a methanol extract exhibited weak antibacterial activity, with a MIC > 512 mg/mL [69]. Rovná et al. reported a MIC of 32 μg/mL for the methanolic extract [70], a value also observed by Polumackanycz et al. for the aqueous rosehip extract [52]. Notably, Miljković et al. found that a 70% ethanol extract exhibited a significantly low MIC of just 4 mg/mL [55]. Wang et al. demonstrated that methanolic or aqueous extracts of *Rosa canina* L. fruit showed good inhibitory activity against several microbes, but when combined with conventional antibiotics significantly enhanced their effectiveness in inhibiting the growth of *Proteus vulgaris*, *Klebsiella pneumoniae*, and *Acinetobacter baylyi*—bacteria associated with rheumatoid arthritis, ankylosing spondylitis, and multiple sclerosis, respectively [71].

#### 3.5.2. Antioxidant Activity

**Figure 6:**
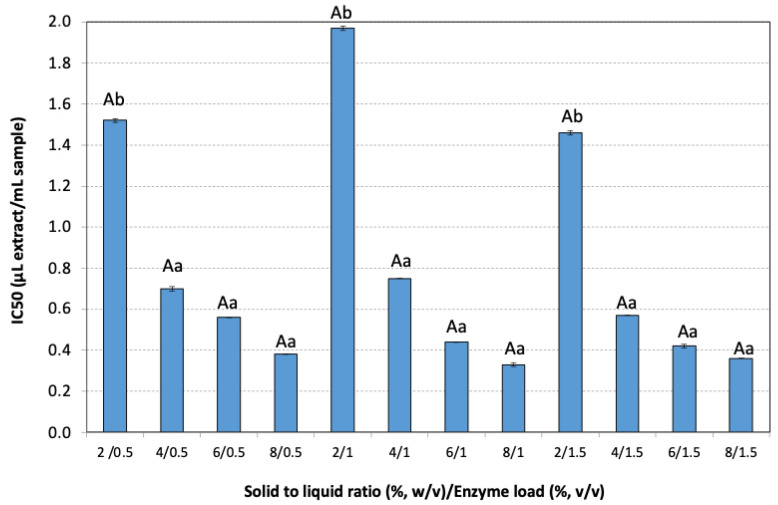
The antioxidant activity (IC_50_) of the different extracts of the pseudo-fruit of *Rosa canina* L. The data are expressed as mean values ± SD and were analyzed using ANOVA to assess the effect of each factor, followed by Tukey’s HSD post-hoc analysis test. The superscript letters indicate significant differences (*p* < 0.05) within the levels of each specific factor. Lowercase letters are used to denote differences within the factor solid to liquid ratio, and uppercase letters are used to denote differences within the enzyme load. Values with different superscript letters are significantly different from each other (*p* < 0.05).

The positive health effects of phenolic compounds are associated with their antioxidant properties, which contribute to protection against chronic diseases such as heart disease and cancer by neutralizing reactive oxygen species like superoxide anions, hydroxyl radicals, and peroxy radicals. The antioxidant activity can be measured by many different methods, such as the 2,2-diphenyl-1-picrylhydrazyl (DPPH), the ferric ion reducing antioxidant power (FRAP), the 2,2′-azino-bis(3-ethylbenzothiazoline-6-sulfonic acid (ABTS), and the cupric ion reducing antioxidant capacity (CUPRAC) methods [55]. In the present study, the antioxidant activity of several extracts was assessed with the DPPH method and expressed as IC_50_ values (Figure 6).

The lowest SLR maintained high IC_50_ values (low antioxidant activity) even with increased enzyme loadings, indicating inefficient extraction. Higher SLRs led to significantly stronger antioxidant activity (lower IC_50_) (*p* < 0.01). Moderate to high enzyme loadings (1–1.5% *v*/*v*), combined with high SLRs consistently showed low IC_50_ values, confirming enhanced extraction efficiency. The highest antioxidant activity (IC_50_ = 0.33 μL extract/mL sample) was obtained at 8% *w*/*v* rosehip concentration and 1% (*v*/*v*) enzyme loading.

Pearson’s correlation analysis was performed in order to investigate the relationship between antioxidant activity and polyphenols (TPC and TFC). Negative correlations were observed between IC_50_ values and polyphenols (both TPC and TFC). More specifically, a moderate negative correlation (r = −0.636, *p* < 0.05) was observed between TPC and IC_50_ values, while a very strong negative correlation (r = −0.786, *p* < 0.05) was noticed between TFC and IC_50_ values, indicating that higher TPC or TFC values are associated with lower IC_50_ values (stronger antioxidant activity). A high polyphenolic content typically leads to increased antioxidant activity in the extract, but factors such as the structure of the polyphenolic compound and the synergistic interactions between polyphenols and other compounds present in the extract play an important role in antioxidant activity.

Moldovan et al. reported the inhibitory activity of the DPPH radicals as 29 mg Trolox per g dw lyophilized extract [50], while Kayahan et al. reported around 95 mg gallic acid/g dw [11]. Liaudanskas et al. examined different species and cultivars of the pseudo-fruit and observed variability across the antioxidant activity of the extracts in a range of 188–397 μmol Trolox/g [47]. It is worth mentioning that *R. canina* fruit extract has been used as a natural antioxidant in mayonnaise [55].

#### 3.5.3. Anti-Aging Activity (Tyrosinase Inhibition)

Melanin is a natural pigment responsible for determining the color of the eyes, hair, and skin. Excessive melanin production can result in conditions such as age spots or melanoma. Tyrosinase (EC 1.14.18.1), a copper-dependent enzyme, is a key enzyme that controls the production of melanin by catalyzing the hydroxylation of L-tyrosine to L-DOPA and the conversion of 3,4-dihydroxyphenylalanine into dopaquinones, followed by polymerization, to form melanin [72]. The inhibition of tyrosinase can reduce the production of melanin and achieve skin whitening, effectively solving pigmentation. Several natural compounds have been found to inhibit tyrosinase activity. Among them, various phenolic compounds—including simple phenols, polyphenols, flavonoids (such as flavones, isoflavones, flavanones, flavanols, dihydroflavones, and anthocyanidins), and tannins—have been widely recognized for their tyrosinase inhibitory effects [73]. These compounds inhibit tyrosinase through multiple mechanisms. Compounds with ortho-dihydroxy groups (e.g., caffeic acid and catechins) chelate copper ions essential for enzyme activity, thereby preventing the oxidation of L-tyrosine to dopaquinone. Additionally, some compounds may act as competitive inhibitors by mimicking natural substrates or be oxidized into reactive quinones that irreversibly modify the enzyme. Hydrophobic interactions and π-π stacking further stabilize the enzyme–inhibitor complex, enhancing inhibition. These multifaceted actions underscore the potential of plant phenolics as therapeutic agents for hyperpigmentation disorders [69].

**Figure 7:**
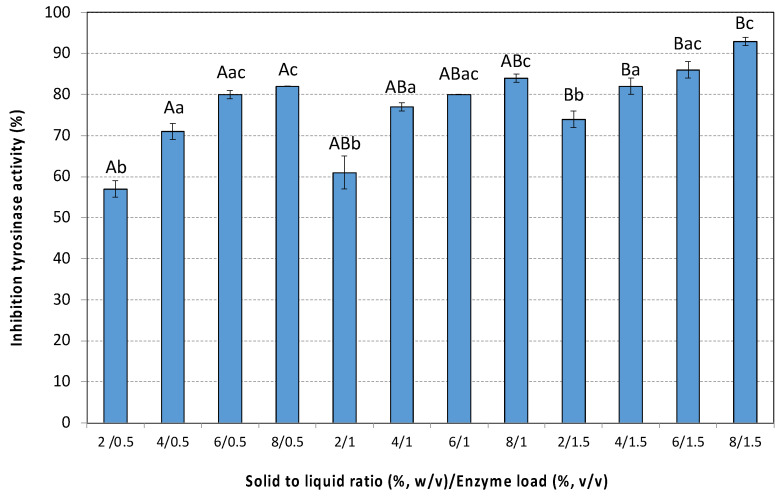
The anti-aging activity (inhibition of tyrosinase activity) of the extracts of the pseudo-fruit of *Rosa canina* L. The data are expressed as mean values ± SD and were analyzed using ANOVA to assess the effect of each factor, followed by Tukey’s HSD post-hoc analysis test. The superscript letters indicate significant differences (*p* < 0.05) within the levels of each specific factor. Lowercase letters are used to denote differences within the factor solid to liquid ratio, and uppercase letters are used to denote differences within the enzyme load. Values with different superscript letters are significantly different from each other (*p* < 0.05).

As shown in Figure 7, all extracts exhibited high tyrosinase inhibition. Kojic acid, a known tyrosinase inhibitor, completely inhibits tyrosinase at a concentration of 1 mg/mL. As SLR increases, the resulting extracts exhibited increased inhibition of tyrosinase activity. This trend is the same at all enzyme loadings tested. Among SLRs, significant differences were observed at 8% *w*/*v* in comparison to the lowest ones (*p* < 0.001). Increasing the enzyme loading from 0.5% to 1.5% (*v*/*v*) significantly enhanced inhibition, especially at low solid to liquid ratios (*p* < 0.01). The highest inhibition (~93%) was observed by the extract obtained at 8% *w*/*v* and 1.5% *v*/*v* enzyme loading.

Strong positive correlations were observed between the inhibition of tyrosinase activity and both TPC and TFC, as indicated by Pearson’s correlation analysis. More specifically, a strong positive correlation was observed between TPC (r = 0.7015, *p* < 0.05) or TFC (r = 0.7515, *p* < 0.05) and inhibition of tyrosinase activity, indicating that higher TPC or TFC values are linked to higher inhibition of tyrosinase. As antioxidant activity increases, inhibition of tyrosinase also improves, indicating a very strong negative correlation (r = −0.8619, *p* < 0.05).

The potential anti-aging effect of *Rosa canina* L., particularly due to inhibiting tyrosinase activity, has also been documented in the literature. An in vivo study by Fujii & Saito demonstrated that quercetin isolated from *Rosa canina* L. reduced melanin synthesis in B16 melanoma cells by inhibiting tyrosinase activity, without affecting cell viability, while oral administration of rosehip extracts decreased skin pigmentation in guinea pigs [74]. A clinical trial conducted by Phetcharat et al. showed that *Rosa canina* L. powder reduced skin wrinkles, and improved moisture and elasticity [75]. An in vitro study by Stankovic et al. investigated tyrosinase inhibition using two different *Rosa canina* L. extracts [76]. These extracts were obtained through ultrasonic treatment with ethanol–water (7:3, *v*/*v*) or propylene glycol–water (45:55, *v*/*v*) as solvents, both of which achieved 50% tyrosinase inhibition. Extracts obtained from aqueous extraction of the rosehips resulted in 72.7% tyrosinase inhibition [77].

#### 3.5.4. Anti-Diabetic Activity (α-Amylase and α-Glucosidase Inhibition)

High levels of blood glucose are characteristic of a group of metabolic diseases called diabetes mellitus. There are three main types of diabetes. Insulin-dependent diabetes (type 1 diabetes, T1D) is a chronic, autoimmune process, characterized by the destruction of pancreatic β-cells by the body’s immune system, which causes the loss of the ability to produce insulin. Non-insulin-dependent diabetes (type 2 diabetes, T2D) is characterized primarily by reduced insulin receptor sensitivity, and gestational diabetes (GD) occurs during pregnancy. T1D and T2D are both chronic conditions and, in conjunction with oxidative stress, a major problem observed during diabetes, can cause secondary ailments such as neuropathy, retinopathy, angiopathy, and nephropathy [78]. According to the International Diabetes Federation (IDF), the global population of people with diabetes mellitus was estimated at 463 million in 2019, and this is projected to rise to 700 million by 2045 if current trends continue [79].

Inhibiting carbohydrate-digesting enzymes like α-amylase and α-glucosidase is an effective therapeutic strategy for managing and treating T2D. α-Amylase (EC 3.2.1.1) is the most essential digestive enzyme, and it catalyzes the hydrolysis of α-1,4 glycosidic bonds in adjacent glucose units of starch results in maltose, while α-glucosidase (EC 3.2.1.20) further degrades maltose to glucose. The inhibitors of α-amylase reduce blood glucose by inhibiting the hydrolysis of the starch, contributing to the improvement in T2D symptoms, while the inhibitors of α-glucosidase delay carbohydrate digestion, limiting glucose absorption and maintaining blood sugar levels [78]. Tannic acid was used in the present study at a concentration of 1 mg/mL as a positive control, since it is a potent inhibitor of both α-glucosidase and α-amylase activity, demonstrating a superior inhibitory effect compared to acarbose, a standard anti-diabetic drug [80].

The pseudo-fruit of *Rosa canina* L. is considered an effective medicinal plant against diabetes mellitus in Iranian and Turkish traditional medicine [81]. Several secondary metabolite groups, including flavonoids, phenolic acids, terpenoids, tannins, alkaloids, xanthones, and polysaccharides have been identified as prospective inhibitors of the α-amylase enzyme [78].

**Figure 8:**
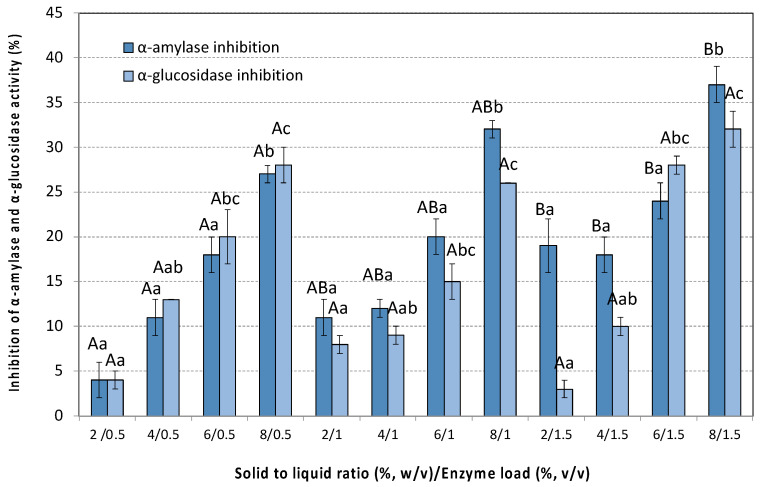
Anti-diabetic activity (inhibition of α-glucosidase and α-amylase activity) by different extracts of the pseudo-fruit of *Rosa canina* L. The data are expressed as mean values ± SD and were analyzed using ANOVA to assess the effect of each factor, followed by Tukey’s HSD post-hoc analysis test. The superscript letters indicate significant differences (*p* < 0.05) within the levels of each specific factor. Lowercase letters are used to denote differences within the factor solid to liquid ratio, and uppercase letters are used to denote differences within the enzyme load. Values with different superscript letters are significantly different from each other (*p* < 0.05).

The inhibitory effect of the extracts on a-amylase activity is presented in Figure 8. All extracts were tested at a concentration of 50 mg/mL. Complete inhibition (100%) of α-amylase activity was achieved in the presence of 1 mg/mL tannic acid. The results of the present study showed moderate to low α-amylase inhibition (Figure 8).

More specifically, extracts obtained from low SLRs exhibited weak inhibitory effects ranging from 1 to 18%, while at high SLRs the inhibitory effect increased. The highest inhibition of a-amylase activity (37 ± 2%) was achieved at a solid to liquid ratio of 8% (*w*/*v*) and an enzyme loading of 1.5% (*v*/*v*).

As in the case of tyrosinase inhibition, strong positive correlations were observed between the inhibition of a-amylase activity and TPC (r = 0.784, *p* < 0.05) or TFC (r = 0.740, *p* < 0.05), as indicated by Pearson’s correlation analysis, suggesting that polyphenolic-rich extracts also exhibit strong α-amylase inhibition. Furthermore, a moderate negative correlation (r = −0.668, *p* < 0.05) was observed between antioxidant activity and the inhibition of a-amylase activity. Jemaa et al. reported that a *Rosa canina* L. extract (10% *w*/*v*, methanol solvent, 24 h extraction time) inhibited 100% of a-amylase activity at a concentration of 5.5 mg/mL [82]. A tisane prepared from *R. canina* rosehip and dried at 60 °C exhibited a 75.51% inhibition of a-amylase, preventing CNP-G3 hydrolysis and the release of the 2-chloro-4-nitrophenol (CNP), a colored by-product [83]. Furthermore, solvent-fractionated ethanolic extracts from *Rosa canina* L. fruits, rich in monosaccharides, oligosaccharides, and pectins, demonstrated notable anti-diabetic effects when administered to Streptozotocin-induced diabetic rats [84]. Also, Taghizadeh et al. [85] showed that rosehip ethanolic extracts obtained from cultivated *R. canina* significantly decreased fasting blood glucose levels in Streptozotocin-induced diabetic rats.

As illustrated in Figure 8, the inhibition of α-glucosidase activity by the studied extracts (at a concentration of 1 mg/mL) followed a similar trend to that observed for α-amylase. Similarly to α-amylase, α-glucosidase activity was completely (100%) inhibited in the presence of 1 mg/mL tannic acid. The highest inhibition of α-glucosidase activity (32 ± 2%) was achieved at an SLR of 8% (*w*/*v*) and an enzyme loading of 1.5% (*v*/*v*).

Pearson’s correlation analysis indicated a strong positive correlation between the inhibition of α-glucosidase activity and TPC of the extract (r = 0.8277 for TPC and r = 0.7157 for TFC, *p* < 0.05), while higher antioxidant activity (lower IC_50_ value) is associated with increased α-glucosidase inhibition (strong negative correlation r = −0.7593, *p* < 0.05).

In vitro studies by Moldovan et al. [50] showed that rosehip extract (8 mg/mL) achieved 15% inhibition of mammalian α-glucosidase, while Asghari et al. showed that different acetone extract fractions displayed 7.9% to 91.2% inhibition in α-glucosidase activity [86]. Plant phenolic compounds inhibit α amylase and α glucosidase via both competitive and allosteric mechanisms. Their hydroxyl groups form hydrogen bonds with active site residues, impeding substrate access, while hydrophobic and π–π stacking interactions stabilize the enzyme–inhibitor complex. This stabilization may induce conformational changes that further reduce catalytic efficiency. Consequently, the enzymatic breakdown of complex carbohydrates is slowed, potentially mitigating postprandial hyperglycemia—a key target in the management of type 2 diabetes [87].

## 4. Conclusions

This study demonstrated that enzyme-assisted extraction (EAE) is a highly efficient and environmentally sustainable approach for extracting phenolic and flavonoid compounds from the pseudo-fruit of *Rosa canina* L. EAE yielded significantly higher TPC and TFC compared to previous studies that applied conventional and non-conventional extraction methods. The power law model was the most adequate of the investigated models in the present study for the description of both TPC and TFC extraction with the aid of the cellulolytic enzymes. The biological activities of the extracts were evaluated, revealing potent tyrosinase inhibition, moderate to low inhibition of α-amylase and α-glucosidase (depending on TPC content), significant antibacterial activity against *Escherichia coli* growth, and notable antioxidant potential.

To the best of our knowledge, this is the first extensive study on the EAE of bio-active compounds from the pseudo-fruit of *Rosa canina* L. with the use of a cellulolytic enzyme. Future research should integrate in vivo assays (cytotoxicity tests) of the extracts to confirm their safety. Moreover, a detailed phytochemical profiling is necessary to identify specific compounds responsible for the observed biological activities, thereby facilitating potential applications in cosmetics, pharmaceuticals, and nutraceuticals.

## Figures and Tables

**Table 1:** Chemical analysis of the hypanthium of pseudo-fruit of *Rosa canina* L.

Component	Concentration (% *w*/*w*, Dry Basis)
Water-soluble compounds *	40.3 ± 1.2
Crude fat	5.4 ± 0.1
Pectin	11.8 ± 1.9
Cellulose	9.9 ± 0.7
Hemicellulose	10.9 ± 0.3
Crude protein	7.5 ± 0.2
Lignin	9.5 ± 4.4
Ash	2.1 ± 0.2

* Glucose and fructose at concentrations of 2.3 ± 0.4 (%, *w*/*w*, dry basis) and 3.3 ± 0.1 (%, *w*/*w*, dry basis), respectively, were detected in the aqueous rosehip extract by HPLC.

**Table 3:** Kinetic models, constants, and regressed statistical parameters for TPC extraction from hypanthium of *Rosa canina* L. via EAE.

			Solid to Liquid Ratio (%, *w*/*v*)/Enzyme Loading (%, *v*/*v*)											
Model			2/0.5	4/0.5	6/0.5	8/0.5	2/1.0	4/1.0	6/1.0	8/1.0	2/1.5	4/1.5	6/1.5	8/1.5
First order	Constants	C_eq_	122.6	121.1	149.8	155.7	127.1	127.6	151.7	153.8	130.9	127.4	153.5	153.1
		k	9.0 × 10^−2^	3.6 × 10^−2^	7.5 × 10^−2^	7.8 × 10^−2^	12 × 10^−2^	3.5 × 10^−2^	6.6 × 10^−2^	11 × 10^−2^	9.5 × 10^−2^	4.5 × 10^−2^	7.3 × 10^−2^	11 × 10^−2^
	Statistical Parameters	R^2^	0.981	0.940	0.968	0.986	0.985	0.949	0.965	0.978	0.964	0.946	0.970	0.982
		R^2^-adj	0.978	0.933	0.964	0.984	0.983	0.943	0.961	0.976	0.960	0.939	0.967	0.980
		NRMSD (%)	4.45	8.28	5.79	3.86	3.95	7.59	6.06	4.71	6.11	7.71	5.57	4.27
Power law	Constants	B	109.4	70.8	113.2	136.2	112.8	71.8	106.2	135,8	101.8	82.6	116.2	135.2
		n	2.0 × 10^−2^	9.0 × 10^−2^	5.1 × 10^−2^	2.3 × 10^−2^	2.2 × 10^−2^	9.7 × 10^−2^	6.4 × 10^−2^	2.3 × 10^−2^	4.6 × 10^−2^	7.5 × 10^−2^	5.0 × 10^−2^	2.3 × 10^−2^
	Statistical Parameters	R^2^	0.981	0.942	0.986	0.983	0.989	0.958	0.994	0.982	0.984	0.959	0.987	0.986
		R^2^-adj	0.979	0.935	0.985	0.981	0.988	0.953	0.993	0.980	0.982	0.954	0.985	0.985
		NRMSD (%)	4.41	8.21	3.76	4.17	3.30	6.95	2.55	4.25	4.09	6.72	4.72	3.71
Peleg’s	Constants	K_1_	2.0 × 10^−2^	1.1 × 10^−2^	3.6 × 10^−2^	2.1 × 10^−2^	3.6 × 10^−2^	1.1 × 10^−2^	4.6 × 10^−2^	1.2 × 10^−2^	3.5 × 10^−2^	7.9 × 10^−2^	3.6 × 10^−2^	1.4 × 10^−2^
		K_2_	8.0 × 10^−3^	7.9 × 10^−3^	6.5 × 10^−3^	6.3 × 10^−3^	6.5 × 10^−3^	7.5 × 10^−3^	6.3 × 10^−3^	6.4 × 10^−3^	7.4 × 10^−3^	7.5 × 10^−3^	6.3 × 10^−3^	6.4 × 10^−3^
		C_eq_	125.0	126.6	153.8	158.7	153.8	133.3	158.7	156.3	135.1	133.3	158.7	156.3
	Statistical Parameters	R^2^	0.982	0.954	0.981	0.986	0.981	0.968	0.986	0.980	0.977	0.965	0.983	0.985
		R^2^-adj	0.980	0.948	0.979	0.984	0.979	0.964	0.984	0.977	0.974	0.960	0.981	0.983
		NRMSD (%)	4.31	7.30	4.40	5.61	17.48	6.07	3.86	4.56	4.86	6.22	4.18	3.95
Second order	Constants	C_e_	124.3	126.9	154.9	158.2	129.2	134.2	158.3	158.2	135.2	133.2	158.7	155.2
		k	3.2 × 10^−3^	5.8 × 10^−4^	1.2 × 10^−3^	2.0 × 10^−3^	3.5 × 10^−3^	5.1 × 10^−4^	8.6 × 10^−4^	2.0 × 10^−3^	1.6 × 10^−3^	7.2 × 10^−4^	1.1 × 10^−3^	3.1 × 10^−3^
	Statistical Parameters	R^2^	0.982	0.954	0.981	0.986	0.982	0.968	0.968	0.986	0.977	0.965	0.983	0.985
		R^2^-adj	0.980	0.948	0.979	0.984	0.980	0.964	0.964	0.984	0.974	0.960	0.981	0.983
		NRMSD (%)	4.30	7.22	4.35	3.81	3.47	6.00	3.77	4.76	4.82	6.17	4.15	3.95

**Table 4:** Kinetic models, constants, and regressed statistical parameters for TFC extraction from hypanthium of *Rosa canina* L. via EAE.

			Solid to Liquid Ratio (%, *w*/*v*)/Enzyme Loading (%, *v*/*v*)											
Model			2/0.5	4/0.5	6/0.5	8/0.5	2/1.0	4/1.0	6/1.0	8/1.0	2/1.5	4/1.5	6/1.5	8/1.5
First order	Constants	C_eq_	44.6	48.4	60.4	60.9	44.5	51.7	59.2	58.3	42.9	51.3	57.6	58.7
		k	5.8 × 10^−2^	8.2 × 10^−2^	5.1 × 10^−2^	6.2 × 10^−2^	5.9 × 10^−2^	7.0 × 10^−2^	5.6 × 10^−2^	1.7 × 10^−1^	6.0 × 10^−2^	8.6 × 10^−2^	5.2 × 10^−2^	4.8 × 10^−2^
	Statistical Parameters	R^2^	0.989	0.965	0.911	0.972	0.971	0.980	0.891	0.937	0.994	0.978	0.952	0.928
		R^2^-adj	0.987	0.961	0.900	0.969	0.967	0.977	0.878	0.929	0.993	0.975	0.946	0.919
		NRMSD (%)	3.80	6.04	10.08	8.38	6.17	4.61	11.19	8.22	2.73	4.77	7.36	8.94
Power	Constants	B	37.4	39.7	35.9	50.1	37.5	40.7	38.6	51.8	34.6	43.6	37.1	41.1
		n	2.9 × 10^−2^	3.6 × 10^−2^	9.2 × 10^−2^	3.3 × 10^−2^	2.8 × 10^−2^	4.2 × 10^−2^	7.6 × 10^−2^	2.2 × 10^−1^	3.8 × 10^−2^	2.9 × 10^−2^	7.7 × 10^−2^	6.1 × 10^−2^
	Statistical Parameters	R^2^	0.962	0.972	0.955	0.961	0.943	0.986	0.919	0.942	0.976	0.981	0.975	0.932
		R^2^-adj	0.955	0.969	0.949	0.956	0.933	0.985	0.909	0.935	0.972	0.979	0.971	0.923
		NRMSD (%)	7.08	5.39	7.16	6.44	8.72	3.77	9.64	7.88	5.61	4.36	5.94	8.77
Peleg’s	Constants	K_1_	1.1 × 10^−1^	7.8 × 10^−2^	1.7 × 10^−1^	4.8 × 10^−2^	1.1 × 10^−1^	9.7 × 10^−2^	1.4 × 10^−1^	3.1 × 10^−2^	1.2 × 10^−1^	5.9 × 10^−2^	1.5 × 10^−1^	1.5 × 10^−1^
		K_2_	2.2 × 10^−2^	2.0 × 10^−2^	1.6 × 10^−2^	1.6 × 10^−2^	2.2 × 10^−2^	1.9 × 10^−2^	1.6 × 10^−2^	1.7 × 10^−2^	2.3 × 10^−2^	1.9 × 10^−2^	1.7 × 10^−2^	1.6 × 10^−2^
		Ceq	45.7	49.5	63.7	61.0	45.5	53.2	61.7	59.2	44.1	52.4	60.2	61.0
	Statistical Parameters	R^2^	0.974	0.970	0.935	0.956	0.955	0.987	0.907	0.939	0.985	0.980	0.966	0.943
		R^2^-adj	0.970	0.967	0.927	0.950	0.947	0.985	0.896	0.932	0.983	0.978	0.962	0.936
		NRMSD (%)	5.81	5.56	8.56	8.82	7.78	3.66	10.34	8.04	4.40	4.51	6.43	8.00
Second order	Constants	Ce	45.6	49.5	63.7	61.1	45.4	61.1	61.8	59.2	44.1	52.2	60.1	61.1
		k	4.3 × 10^−3^	5.3 × 10^−3^	1.5 × 10^−3^	5.6 × 10^−3^	4.5 × 10^−3^	5.6 × 10^−3^	1.9 × 10^−3^	9.1 × 10^−3^	4.3 × 10^−3^	6.2 × 10^−3^	1.8 × 10^−3^	1.8 × 10^−3^
	Statistical Parameters	R^2^	0.974	0.970	0.935	0.956	0.955	0.956	0.907	0.939	0.985	0.980	0.983	0.943
		R^2^-adj	0.970	0.967	0.927	0.950	0.947	0.950	0.896	0.932	0.983	0.978	0.981	0.936
		NRMSD (%)	5.81	5.56	8.55	6.82	7.78	17.57	10.34	8.04	4.40	4.51	6.42	8.00

## Data Availability

Data are contained within the article.

## Abbreviations

The following abbreviations are used in this manuscript:

EAE	Enzyme-assisted extraction
TPC	Total Phenolic Content
TFC	Total Flavonoid Content
GAE	Gallic Acid Equivalents
DM	Dry Matter
CAE	Catechin Equivalents
UAE	Ultrasound-Assisted Extraction
MAE	Microwave-Assisted Extraction
NADES	Natural Deep Eutectic Solvent
PEF	Pulsed Electric Field Extraction
MHG	Microwave Hydrodiffusion and Gravity
UAEE	Ultrasound-Assisted Enzymatic Extraction
SFE	Supercritical carbon dioxide extraction
SLR	Solid to liquid ratio
MIC	Minimum Inhibitory Concentration
ANOVA	Analysis of Variance
SD	Standard Deviation
NRMSD	Normalized Root Mean Square Deviation
